# Investigating the Multitarget Pharmacological Mechanism of Ursolic Acid Acting on Colon Cancer: A Network Pharmacology Approach

**DOI:** 10.1155/2021/9980949

**Published:** 2021-06-04

**Authors:** Jun Zhao, Ping Leng, Wen Xu, Jia-Lin Sun, Bei-Bei Ni, Guang-Wei Liu

**Affiliations:** ^1^Department of Pharmacy, The Affiliated Hospital of Qingdao University, Qingdao 266003, Shandong, China; ^2^Department of Gastrointestinal Surgery, The Affiliated Hospital of Qingdao University, Qingdao 266003, Shandong, China

## Abstract

**Objective:**

To explore the mechanisms of ursolic acid for treating colon cancer based on network pharmacology.

**Method:**

In this study, the potential targets of ursolic acid against colon cancer were predicted and screened through the TCMSP, SYMMAP, Drug Bank, UNI-PROT, and DISGENET databases. The protein interaction (PPI) network was constructed based on the STRING database, and graphs were drawn with the help of Cytoscape software. GO and KEGG enrichment analyses were performed on the targets by using the DAVID database for biological information annotation.

**Results:**

Ursolic acid has 113 targets in the treatment of colon cancer. The core targets included interleukin-6 (IL-6), mitogen-activated protein kinase 3 (MAPK3), vascular endothelial growth factor receptor (VEGFA), prostaglandin endoperoxide synthase 2 (PTGS2), caspase-3 (CASP3), mitogen-activated protein kinase 8 (MAPK8), tumor necrosis factor (TNF), cyclin D1 (CCND1), JUN, signal transducer and transcriptional activator 3 (STAT3), and other targets. The first 10 pathways related to colon cancer were screened out. The main signaling pathways included the TNF signaling pathway and the AGE-RAGE signaling pathway in diabetic complications and human colon cancer infections.

**Conclusion:**

This study revealed that ursolic acid played a multitarget and multichannel antitumor role by inhibiting the proliferation of tumor cells, inducing apoptosis, and enhancing antiangiogenesis.

## 1. Introduction

Colon cancer is a common malignant tumor of the digestive tract, which seriously threatens human health. Natural Chinese medicine has shown antitumor potential through a “multicomponent, multitarget, and multipathway” approach. Furthermore, researchers have been attracted by traditional Chinese medicine's low cost, stable curative effect, and minimal side effects, leading them to explore TCM's potential and application prospects for treating tumors. Ursolic acid (UA) is a natural compound with biological characteristics that can inhibit tumor cell proliferation, induce apoptosis, resist mutation, resist oxidation, and resist angiogenesis. Due to Chinese medicine's promising benefits in tumor prevention and treatment, it has become a major focus of research in recent years. Since the mechanism of ursolic acid's antitumor effect is complex, its targets and molecular mechanism for treating colon cancer have not been fully understood by scholars at this stage. In this study, the related mechanisms of ursolic acid against colon cancer were predicted using a network pharmacology research method, which provided the basis to further understand the antitumor mechanisms of ursolic acid.

## 2. Materials and Methods

### 2.1. Extraction of Ursolic Acid Targets

The monomer compounds of ursolic acid were queried in the TCMSP (Traditional Chinese Medicine Systems Pharmacology Database and Analysis Platform, https://tcmspw.com/tcmsp.php), the SwissTargetPrediction database (http://www.swisstargetprediction.ch/), and the BATMAN-TCM database (Bioinformatics Analysis Tool for Molecular Mechanism of Traditional Chinese Medicine, http://bionet.ncpsb.org/batman-tcm/). The structures of the compounds were then compared in the PubMed database. The intervention targets of the ursolic acid monomer compounds were queried in the SEA (Similarity Ensemble Approach, http://sea.bkslab.org/) database. The names of the proteins corresponding to the above targets were converted into human gene symbols by the UniProt (https://www.uniprot.org/) database. Finally, the database of ursolic acid monomer compounds and their intervention targets was constructed after the duplicates were removed.

### 2.2. Screening of Colon Cancer-Related Targets

Using “Colon cancer” as the key word, human genes were queried in the GeneCards database (https://www.genecards.org/), the NCBI gene database (https://www.ncbi.nlm.nih.gov/), and the OMIM database (https://www.omim.org/). Among them, the data from GeneCards were used to determine the relevance score to obtain more relevant targets.

The two selected targets were input into Venny 2.1, and the Wayne map of ursolic acid and the colon cancer target gene was drawn.

### 2.3. Construction and Analysis of the Protein Interaction Network

Common drug targets and disease targets were input into the STRING database (https://string-db.org/cgi/input.pl). The biological species were set as “Homo sapiens” and the PPI network was constructed. The results were then imported into Cytoscape software to draw the interaction network, and the node size and color depth were set to reflect the degree. The darker the node color and the larger the shape, the greater the “degree” value of the corresponding target protein. The thickness of the line was used to represent the size of the combined score; that is, the thicker the line, the larger the value of the combined score. The PPI network was analyzed after it was completed.

### 2.4. Topology Analysis

The PPI network was imported into Cyber 3.8.0, and a topology analysis was conducted with the Network Analyzer tool using four parameters: (1) degree, (2) betweenness centrality, (3) average shortest path length; and (4) closeness centrality as reference standards. According to the degree sorting procedure, genes with scores greater than the average score were selected as key targets, the first 20 targets were drawn with R 3.6.3, and the abscissa represented the degree value of each target.

### 2.5. Survival Analysis

The information for these gene survival analyses comes from the OncoLnc database (http://www.oncolnc.org/). It contains RNA-SEQ expression and survival data of 8647 patients' mRNAs and miRNAs in 21 cancer studies, which are conducted by the Cancer Genome Atlas (TCGA).

### 2.6. GO Enrichment Analysis

The common drug targets and disease targets were enriched by biological process (BP), molecular function (MF), and cell component (CC) of GO. The items with corrected *P* value ≤0.05 were screened using the STRING database to obtain the intersection target enrichment data. Using the R 3.6.3 software, the cluster profiler was installed and a quote was obtained, the plot was enriched, and the ggplot2 package was used to draw histograms and bubble charts.

### 2.7. KEGG Pathway Enrichment

The KEGG pathway enrichment analysis was carried out on common drug and disease targets, and the items with corrected *P* value ≤0.05 were screened using the STRING database to obtain pathway enrichment data. Using the R 3.6.3 software, the cluster profiler package was installed and referenced, and histograms and bubble charts were drawn.

### 2.8. Ursolic-Acid Key-Target Main-Pathway Colon Cancer Network Construction

The ursolic-acid key-target main-pathway colon cancer network file was imported into Cytoscape 3.8.0 to draw the pathway network diagram. With the pathway network diagram, it was possible to achieve a clearer understanding of the multitarget action characteristics of ursolic acid in the treatment of colon cancer.

## 3. Results

### 3.1. Ursolic Acid and Colon Cancer Target Screening Results

After searching, summarizing, and deleting duplicates, a total of 172 targets were obtained for ursolic acid. 3,112 colon cancer-related genes were retrieved from the GeneCards database, 1,518 colon cancer-related genes were obtained from the NCBI database, and 500 colon cancer-related targets were obtained from the OMIM database. After merging and deleting the duplicate genes from these three databases, 4,024 colon cancer-related genes were obtained.

**Figure 1:**
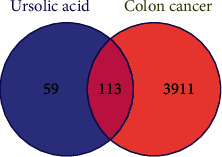
Venn diagram of potential targets of ursolic acid and colon cancer.

The screened targets were input into Venny 2.1, and 113 common targets were obtained (Figure 1).

### 3.2. PPI Network and Network Topology Analysis Results

**Figure 2:**
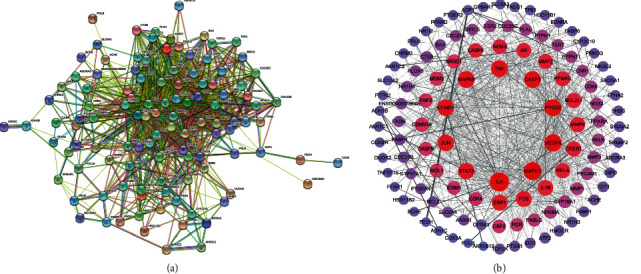
PPI analysis of intersection targets between ursolic acid and colon cancer related targets.

The ursolic acid and colon cancer disease intersection targets were imported into the STRING database, and the biological species was set as “Homo sapiens”. The protein interaction relationship was obtained, the TSV file was saved, and the network visualization was constructed with the help of Cytoscape software (Figure 2). The results showed that there were 113 nodes in the network interacting through 1,179 edges, and the protein interaction was enriched (*P* < 0.001), with an average local clustering coefficient of 0.605.

**Figure 3:**
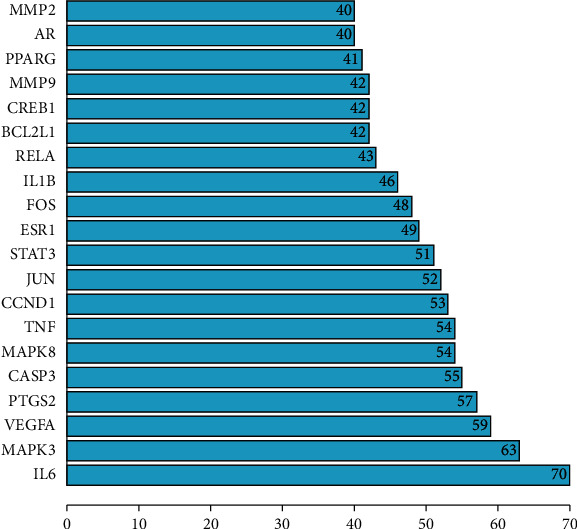
The top 20 significant targets for intersection targets between ursolic acid and colon cancer.

When the “degree” value of a target protein exceeds the average value of the network, it indicates that the target protein is the core protein of the PPI network. In this study, the key target was for the score to be greater than the network average (degree ≥20.9). Topological analysis showed that 44 key targets were screened out. The higher the degree, the greater the role it played in the whole network. The top 20 targets were sorted by degree value and the key gene nodes were mapped accordingly, as shown in Table 1 and Figure 3. The key targets mainly included interleukin-6 (IL-6), mitogen-activated protein kinase 3 (MAPK3), vascular endothelial growth factor receptor (VEGFA), prostaglandin endoperoxide synthase 2 (PTGS2), caspase-3 (CASP3), mitogen-activated protein kinase 8 (MAPK8), tumor necrosis factor (TNF), cyclin D1 (CCND1), JUN and signal transducer and transcription activator 3 (STAT3). These protein genes with higher degrees played a key role in the whole network. The targets corresponding to these protein genes may be the potential key targets of ursolic acid in the treatment of colon cancer.

### 3.3. Survival Analysis

**Figure 4:**
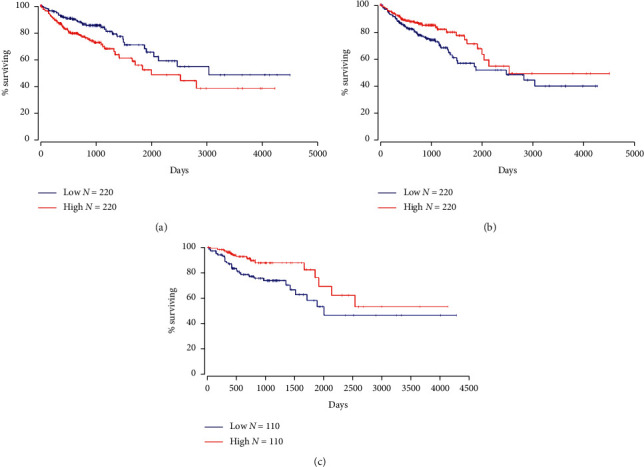
Survival analysis of part of target genes (*P* < 0.05). (a) VEGFA (*P* value = 0.0072). (b) PTGS2 (*P* value = 0.0357). (c) CASP3 (*P* value = 0.0154).

To clarify the impact of these genes on survival and prognosis of colon cancer, survival analysis was performed. The results of *P* < 0.05 are shown in Figure 4. The results indicated that some genes play an important role in the colon cancer patients' survival and prognosis, including VEGFA, PTGS2, and CAPS3. The result suggested that ursolic acid could act on these genes to improve the prognosis of colon cancer.

### 3.4. GO and KEGG Enrichment Analysis Results

**Figure 5:**
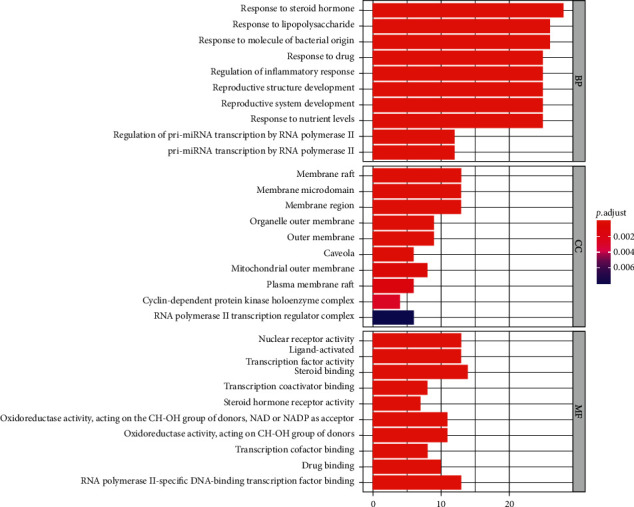
Histogram of GO functional enrichment analysis.

**Figure 6:**
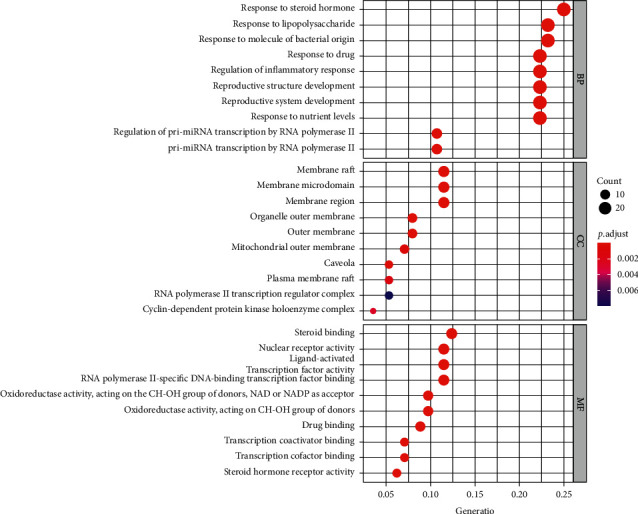
Bubble diagram of GO functional enrichment analysis.

GO biological function enrichment analysis includes biological process (BP), cellular component (CC), and molecular function (MF). The items with corrected *P* value ≤0.05 were screened, and 1,716 GO items were obtained by GO enrichment analysis, including 1,579 BP, 20 CC, and 126 MF. The top 10 items of each module were selected according to the *P* value, as shown in Figures 5 and 6. Biological process analysis showed that the targets mainly responded to steroid hormones, lipopolysaccharides, molecules of bacterial origin, and biological processes such as the response to drugs and regulation of inflammation. The cell components analysis showed that these targets were mainly involved in the molecular composition of membrane rafts, membrane microdomains, membrane regions, organelle outer membranes, and outer membranes. Molecular functional analysis showed that the targets were mainly affected by nuclear receptor activity, ligand-activated transcription factor activity, steroid binding, transcription coactivator binding and steroid hormone receptor activity, and other molecular functions.

**Figure 7:**
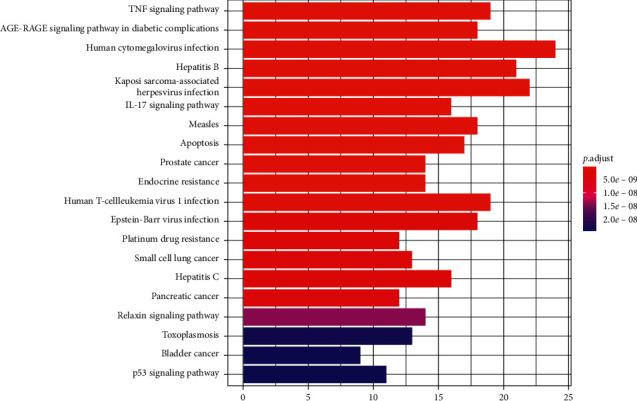
The top 20 significant enriched KEGG pathways for intersection target (histogram).

**Figure 8:**
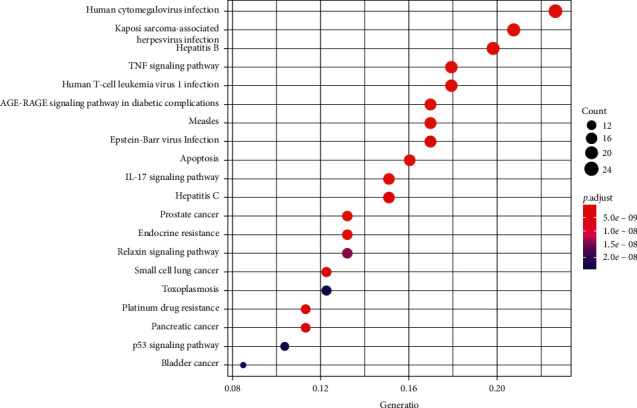
The top 20 significant enriched KEGG pathways for intersection target (bubble diagram).

The items with corrected *P* value ≤0.05 were screened, and 131 signal channels were obtained by KEGG channel enrichment analysis. The top 20 items were selected according to the *P* value, and the KEGG channel enrichment analysis histogram and bubble chart were drawn, as shown in Figures 7 and 8. The results showed that the targets of ursolic acid in treating colon cancer mainly included the TNF signaling pathway; the AGE-RAGE signaling pathway in diabetic complications, human cancer virus infections, hepatitis B, and Kaposi sarcoma-associated virus infections; and IL-17 signaling pathways. See Table 2 for details.

### 3.5. Ursolic-Acid Key-Target Main-Pathway Colon Cancer Network Construction

**Figure 9:**
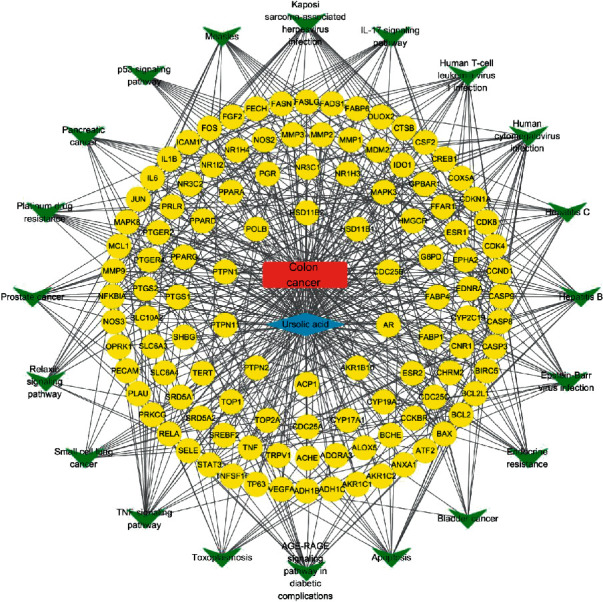
Ursolic-acid key-target main-pathway colon cancer network. Note: as shown in the figure, blue represented the ursolic acid compound, yellow represented the targets of traditional Chinese medicine components acting on the disease, green represented the most significant first 20 pathways, and red represented the disease, namely colon cancer.

Compounds and target points corresponding to the compounds and key target points screened in the experiments were introduced into Cytoscape to construct the network, and the results are shown in Figure 9. It was observed that ursolic acid played a role in the treatment of colon cancer through multitarget action and regulation of multiple signal pathways.

## 4. Discussion

Colon cancer is one of the most common malignant tumors, and among malignant tumors, its incidence ranks third and its mortality ranks second in the world. Within the digestive system, it is the malignant tumor that has the highest incidence and mortality in the world, seriously threatening people's lives and health [1]. At present, the common clinical treatments for colon cancer include surgical resection, radiotherapy, chemotherapy, and molecular targeted therapy. In addition, traditional Chinese medicine has played an important role in the treatment due to its low toxicity, effectiveness, and low cost. Therefore, it is of great clinical value to actively explore the role of natural Chinese medicine in antitumor treatment.

Ursolic acid (UA, 3*β*-hydroxyurs-12-en-28-OIC acid), a pentacyclic triterpene compound, is widely found in natural medicines such as bear fruit, *Ligustrum lucidum*, *Rosa multiflora*, *Hedyotis diffusa*, *Plantago asiatica*, and hawthorn. These drugs are often used to treat colon cancer in China. Through in-depth research on the antitumor properties of ursolic acid, studies have shown that UA regulated cell transcription factors, growth factor receptors, inflammatory cytokines, and other major molecular targets through various mechanisms and signal pathways, and thereby regulated the proliferation, apoptosis, metastasis, autophagy, and angiogenesis of cancer cells [2, 3]. Ursolic acid also has broad spectrum antitumor properties, low toxicity, and minimal side effects on normal cells. As such, its antitumor effect has attracted increasing attention, making it an ideal candidate for the natural treatment of tumors.

In this study, the drug-disease-target network was constructed using the network pharmacology research method, and the GO function and KEGG signaling pathways of the key targets were enriched and analyzed, in order to systematically explore the mechanisms of ursolic acid in treating colon cancer. The results of the PPI network showed that the key targets of ursolic acid in the treatment of colon cancer mainly included interleukin-6 (IL-6), mitogen-activated protein kinase 3 (MAPK3), vascular endothelial growth factor receptor (VEGFA), prostaglandin endoperoxide synthase 2 (PTGS2/COX2), caspase-3 (CASP3), mitogen-activated protein kinase 8 (MAPK8), tumor necrosis factor (TNF), cyclin D1 (CCND1), JUN, and signal transducer and transcription activator 3 (STAT3).

The microenvironment of tumor inflammation is a research topic that has attracted a great deal of attention from scholars in China and abroad. Some scholars call tumor-related inflammation the seventh feature of tumors [4]. The inflammatory microenvironment can impact the occurrence and development of tumors. Tumor necrosis factor, interleukin family, and chemokine family are cytokines that are currently heavily researched for their roles in the regulation of inflammatory response. Many key targets screened in this study, such as IL-6, TNF, and COX2, belong to this category.

IL-6 and IL-17 can damage the body's immune cells (such as Th1 cells and macrophages), which are destroyed to promote the occurrence and development of tumors [5]. IL-6 can induce the activation of three members of the JAK family, namely the signal transducer and activator of transcription 3 (STAT3), mitogen-activation protein kinase (MAPK), and phosphatidylinositol 3-kinase (phosphatidylinositol 3-kinase, PI3K). These three factors are closely related to the occurrence and development of tumors. Three classical inflammatory pathways: MAPKs, IL-6/STAT3, and PI3K were downregulated by UA treatment [6]. Wang et al. found that ursolic acid can reduce the expression of IL-6 to inhibit the activation of the STAT3 signaling pathway in tumor cells and induce tumor cell apoptosis. It can also induce the lysis of colon cancer stem cells, caspase-3, by inhibiting the phosphorylation of STAT3 [7].

Studies have confirmed that IL-17 can induce a huge expansion of MDSCs around tumor tissues, inhibit the infiltration of CD8 + T cells, and enhance immunosuppressive effects [8]. Ursolic acid may suppress the MDSC-mediated immunosuppressive effect by inhibiting the expansion of MDSCs in the microenvironment and enhancing the body's immune response to kill tumor cells. In this way, ursolic acid regulates the tumor immune microenvironment and aids the immune system to kill colon cancer cells.

TNF-*α* is an important proinflammatory factor in the inflammatory microenvironment of a tumor, which can induce target cell DNA mutations, malignant cell proliferation, and neovascularization [9]. Bhat et al. [10] found that TNF-*α* acted on the colon cancer cells to promote their invasion and metastasis. In addition, TNF-*α* increased the expression of PD-L1 in colon cancer, leading to tumor immunosuppression [11].

Angiogenesis is the key to tumor growth, invasion, and metastasis, and it provides nutritional support for tumor growth. Inhibiting tumor angiogenesis is an entirely new, targeted therapy strategy that can control and kill the greatest amount of tumors. VEGFA belongs to the VEGF family and is an important factor in the promotion of tumor angiogenesis [12]. Hypoxia-inducible factor-1*α* (HIF-1*α*), a key mediator in tumor metastasis and angiogenesis, is associated with poor patient prognosis and has been recognized as an important cancer drug target [13]. Wu et al. [14] found that ursolic acid and its derivatives downregulated the expression of HIF-1*α* protein, reduced the production of vascular endothelial growth factor by inhibiting its synthesis, and inhibited the proliferation of colon cancer cells. Ursolic acid can inhibit the expression of newborn VEGF and inhibit the accumulation of HIF-1*α* and the expression of the MDR1 gene and protein under hypoxia. It has a sensitizing effect on chemotherapy of colon cancer cells [15].

The caspase family is a key factor in the process of regulating cell apoptosis. Among them, caspase-3 is the most critical apoptotic effector in this family. It is considered to be the executor of apoptosis. If its expression is missing or decreased, cancer cells have a greater chance of escaping apoptosis. The cancer cells' proliferation ability is enhanced, allowing them to further develop, metastasize, and invade [16]. Zheng et al. found that ursolic acid and oxaliplatin can induce apoptosis of colon cancer cells by enhancing the activity of caspase-3, synergistically inhibiting the proliferation of colon cancer cells [17]. In addition, other studies have also found that ursolic acid downregulated the expression of Bcl-2 by activating caspase-3, caspase-8, and caspase-9, thereby inhibiting the proliferation of cancer cells and inducing apoptosis [18]. The studies showed that ursolic acid can induce tumor cell apoptosis by activating caspase-3 to achieve a certain antitumor effect.

KEGG enrichment pathway analysis mainly involves multiple signaling pathways such as the TNF signaling pathway, the AGE-RAGE signaling pathway in diabetic complications, human cytomegalovirus infections, hepatitis B and Kaposi sarcoma-associated herpesvirus infections, and the IL-17 signaling pathway.

Previous studies have found that the AGE/RAGE signaling pathway activated the downstream HIF-1a and PI3K/AKT signaling pathways in the cell and then promoted tumor cell proliferation, migration, invasion, cloning, and spheroidization, which inhibited cell apoptosis and activated the EMT process [19]. EMT plays an important role in the occurrence and development of colorectal cancer. It is involved in tumor budding, invasion, distant metastasis, angiogenesis, and drug resistance by activating multiple signal pathways. Related studies suggested that the antiproliferation and antimetastasis activities of UA were through EMT inhibition in colorectal cancer [3]. The most important point to note is that colorectal cancer cells can acquire the colon cancer stem cell (colorectal cancer stem cells, CSCs) phenotype through the activation of intracellular EMT. CSCs are a subgroup of tumor cells with high self-renewal ability and they are highly resistant to antitumor drugs and radiotherapy [20]. In addition, studies have found that the continuous activation of RAGE in cells activated the mitogen-activated protein kinase (MAPK) signaling pathway, which recruited IKK in the cytoplasm, inhibited I-*κ*B, and then released and activated NF-*κ*B. As the main transcriptional regulatory element of inflammatory response, NF-*κ*B induced the expression of a variety of inflammatory response-related mRNAs, such as COX-2 (cyclo-oxygenase-2, cyclooxygenase-2), TNF-a (tumor necrosis factor *α*, tumor necrosis factor-a), and IL-6 (interleukin-6, interleukin-6) and then continued to upregulate its synthesis and secretion [21]. Current studies have shown that ursolic acid has a regulatory effect on the downstream signaling pathways of this pathway (PI3K/AKT signaling pathway, MAPK signaling pathway, and other inflammatory response pathways).

Human cytomegalovirus (HCMV) infection is a carcinogenic factor discovered in recent years and is closely related to colorectal cancer [22, 23]. Related studies have found that the HCMV signaling pathway can induce the activation of platelet-derived growth factor receptor *α* (PDGFR*α*) and PI3K/AKT in colon cancer cells, thereby promoting the proliferation of cancer cells and enhancing their migration ability [24]. Studies such as that of Wan Huai Teo et al. have found that colon cancer cell proliferation and migration ability increased significantly after HCMV infection, which may be closely related to the Wnt signaling pathway and EMT [25]. In addition, the role of HCMV in promoting STAT3 phosphorylation suggested that HCMV may participate in the occurrence and development of malignant tumors through the IL-6/JAK/STAT3 signaling pathway [26, 27]. Our study found that ursolic acid has an anti-HCMV effect in vitro [28].

## 5. Summary

In summary, after combining the results of this study with literature data, it was found that a possible mechanism for ursolic acid to act against colon cancer was through the regulation of key targets such as IL-6, VEGFA, MAPK, and caspases and through multiple key pathways to inhibit tumor cell proliferation and induce cell apoptosis and antitumor functions such as death and antiangiogenesis. There were multiple therapeutic targets and signal pathways in this process, which reflected the multitarget and multichannel treatment of diseases that are characteristic of traditional Chinese medicine. Regarding the targets and pathways of ursolic acid for colon cancer treatment predicted in this study, the specific mechanism needs to be further verified through experiments, which can provide a certain reference basis for subsequent research on ursolic acid's prevention and treatment of tumors.

## Figures and Tables

**Table 1:** Topological analysis of the targets of ursolic acid on colon cancer (top 20).

Name	Degree	Betweenness centrality	Closeness centrality	Clustering coefficient	Eccentricity	Radiality	Topological coefficient
IL6	70	0.112	0.709	0.337	4	0.932	0.256
MAPK3	63	0.061	0.675	0.391	4	0.920	0.274
VEGFA	59	0.044	0.647	0.437	4	0.909	0.290
PTGS2	57	0.054	0.655	0.435	4	0.912	0.284
CASP3	55	0.025	0.615	0.486	5	0.896	0.316
TNF	54	0.024	0.636	0.481	4	0.905	0.302
MAPK8	54	0.025	0.636	0.488	4	0.905	0.300
CCND1	53	0.045	0.619	0.470	4	0.897	0.307
JUN	52	0.027	0.619	0.506	4	0.897	0.318
STAT3	51	0.033	0.612	0.515	4	0.894	0.323
ESR1	49	0.037	0.609	0.474	4	0.893	0.309
FOS	48	0.023	0.609	0.543	4	0.893	0.329
IL1B	46	0.022	0.609	0.506	4	0.893	0.304
RELA	43	0.018	0.583	0.570	5	0.881	0.342
CREB1	42	0.043	0.589	0.480	5	0.884	0.316
MMP9	42	0.007	0.580	0.649	4	0.879	0.360
BCL2L1	42	0.007	0.577	0.626	4	0.878	0.356
PPARG	41	0.027	0.583	0.479	4	0.881	0.314
MMP2	40	0.009	0.577	0.650	4	0.878	0.359
AR	40	0.037	0.577	0.490	4	0.878	0.323

**Table 2:** The top 20 significant enriched KEGG pathways for intersection target.

Description	*P* value	Count	Gene ID
TNF signaling pathway	1.68*E−*16	19	MAPK3/PTGS2/TNF/MMP3/RELA/FOS/MMP9/JUN/IL6/CASP3/MAPK8/NFKBIA/CASP8/ICAM1/IL1B/CREB1/SELE/ATF2/CSF2
AGE-RAGE signaling pathway in diabetic complications	3.75*E−*16	18	MAPK3/TNF/MMP2/RELA/STAT3/VEGFA/CCND1/BCL2/BAX/JUN/IL6/CASP3/MAPK8/ICAM1/IL1B/SELE/NOS3/CDK4
Human cytomegalovirus infection	7.12*E−*16	24	MAPK3/PTGER2/PTGS2/PTGER4/TNF/MDM2/RELA/STAT3/VEGFA/CCND1/CDKN1A/BAX/CASP9/IL6/CASP3/NFKBIA/CASP8/IL1B/CREB1/PRKCG/ATF2/FASLG/CDK4/CDK6
Hepatitis B	1.09*E−*15	21	MAPK3/TNF/RELA/STAT3/BCL2/FOS/CDKN1A/BAX/CASP9/MMP9/JUN/IL6/CASP3/MAPK8/NFKBIA/CASP8/CREB1/PRKCG/ATF2/BIRC5/FASLG
Kaposi sarcoma-associated herpesvirus infection	3.32*E−*15	22	MAPK3/PTGS2/RELA/STAT3/VEGFA/CCND1/FOS/CDKN1A/BAX/CASP9/JUN/IL6/CASP3/MAPK8/NFKBIA/CASP8/ICAM1/CREB1/CSF2/CDK4/CDK6/FGF2
IL-17 signaling pathway	4.70*E−*14	16	MAPK3/PTGS2/TNF/MMP3/MMP1/RELA/FOS/MMP9/JUN/IL6/CASP3/MAPK8/NFKBIA/CASP8/IL1B/CSF2
Measles	1.51*E−*13	18	RELA/STAT3/CCND1/BCL2/BCL2L1/FOS/BAX/CASP9/JUN/IL6/CASP3/MAPK8/NFKBIA/CASP8/IL1B/FASLG/CDK4/CDK6
Apoptosis	1.38*E−*12	17	MAPK3/TNF/CTSB/RELA/BCL2/BCL2L1/FOS/BAX/CASP9/JUN/CASP3/MAPK8/NFKBIA/CASP8/MCL1/BIRC5/FASLG
Prostate cancer	2.09*E−*11	14	AR/MAPK3/SRD5A2/MMP3/MDM2/PLAU/RELA/CCND1/BCL2/CDKN1A/CASP9/MMP9/NFKBIA/CREB1
Endocrine resistance	2.41*E−*11	14	MAPK3/ESR2/ESR1/MMP2/MDM2/CCND1/BCL2/FOS/CDKN1A/BAX/MMP9/JUN/MAPK8/CDK4
Human T-cell leukemia virus 1 infection	4.25*E−*11	19	POLB/TERT/MAPK3/TNF/RELA/CCND1/BCL2L1/FOS/CDKN1A/BAX/JUN/IL6/MAPK8/NFKBIA/ICAM1/CREB1/ATF2/CSF2/CDK4
Epstein-Barr virus infection	9.41*E−*11	18	TNF/MDM2/RELA/STAT3/CCND1/BCL2/CDKN1A/BAX/CASP9/JUN/IL6/CASP3/MAPK8/NFKBIA/CASP8/ICAM1/CDK4/CDK6
Platinum drug resistance	1.29*E−*10	12	TOP2A/MAPK3/MDM2/BCL2/BCL2L1/CDKN1A/BAX/CASP9/CASP3/CASP8/BIRC5/FASLG
Small cell lung cancer	1.51*E−*10	13	NOS2/PTGS2/RELA/CCND1/BCL2/BCL2L1/CDKN1A/BAX/CASP9/CASP3/NFKBIA/CDK4/CDK6
Hepatitis C	1.55*E−*10	16	NR1H3/PPARA/MAPK3/TNF/RELA/STAT3/CCND1/CDKN1A/BAX/CASP9/CASP3/NFKBIA/CASP8/FASLG/CDK4/CDK6
Pancreatic cancer	2.11*E−*10	12	MAPK3/RELA/STAT3/VEGFA/CCND1/BCL2L1/CDKN1A/BAX/CASP9/MAPK8/CDK4/CDK6
Relaxin signaling pathway	1.03*E−*09	14	MAPK3/NOS2/MMP1/MMP2/RELA/VEGFA/FOS/MMP9/JUN/MAPK8/NFKBIA/CREB1/ATF2/NOS3
Toxoplasmosis	1.85*E−*09	13	MAPK3/NOS2/ALOX5/TNF/RELA/STAT3/BCL2/BCL2L1/CASP9/CASP3/MAPK8/NFKBIA/CASP8
Bladder cancer	2.00*E−*09	9	MAPK3/MMP1/MMP2/MDM2/VEGFA/CCND1/CDKN1A/MMP9/CDK4
p53 signaling pathway	2.10*E−*09	11	MDM2/CCND1/BCL2/BCL2L1/CDKN1A/BAX/CASP9/CASP3/CASP8/CDK4/CDK6

## Data Availability

The data used to support the findings of this study are available from the corresponding author upon request.
